# New Advances in the Genetics and Evolution of Ladybird Beetles for Biological Control

**DOI:** 10.3390/insects16080753

**Published:** 2025-07-23

**Authors:** Hong Pang, Hao-Sen Li

**Affiliations:** State Key Laboratory of Biocontrol, School of Ecology, Sun Yat-sen University, Shenzhen 518000, China

Ladybird beetles (Coccinellidae) have long been recognized as invaluable natural enemies in biological control, owing to their strong ability to suppress aphids, coccids, and other small pests. However, their genetic makeup and evolutionary dynamics—particularly those influenced by artificial rearing and release—impact their pest-control efficacy. Recent studies leveraging modern approaches in genetics, physiology, and predator–prey interactions are deepening our understanding of the functional mechanisms and adaptive capacities of these ladybirds. This Special Issue, “Genetics and Evolution of Ladybird Beetles in Biological Control”, in *Insects* compiles a diverse array of research, exploring how evolutionary processes, breeding strategies, and ecological factors shape the role of ladybirds in pest management. Covering topics from gene function to field performance, this collection offers a broad view of ladybirds as dynamic and adaptable players in modern integrated pest management.

This Special Issue includes two articles exploring the molecular basis of reproduction in *Coccinella septempunctata*—one of the most widely utilized ladybird species in biological control. Feng et al. [Article 1] investigated two insulin-like peptide genes, *CsILP1* and *CsILP2*, demonstrating their crucial role in ovary development and egg production. When these genes were silenced using RNA interference, the expression of key reproduction-related genes declined significantly, accompanied by a marked reduction in female fecundity. This finding identifies valuable targets for enhancing reproductive performance in mass-rearing programs. Similarly, Cheng et al. [Article 2] investigated two additional genes, *Met* and *Kr-h1*, which are involved in juvenile hormone signaling and male reproductive processes. Silencing these genes reduced testis development in males and led to a noticeable decrease in the number of eggs laid by their mates. Together, these studies highlight how internal molecular signals regulate reproductive capacity, a key trait for both wild and commercially reared ladybird populations.

The role of *Harmonia axyridis*—a widely deployed yet sometimes controversial ladybird species—is also featured in this Special Issue. Li et al. [Article 3] investigated the predatory capacity of *H. axyridis* against the water lily aphid (*Rhopalosiphum nymphaeae*), showing that all life stages of the beetle exhibit strong responsiveness to fluctuating prey densities. This finding provides valuable insights for controlling aphid outbreaks in aquatic or wetland crop systems. On the other hand, Fan et al. [Article 4] explored how the presence of *H. axyridis*—even in the absence of actual predation—impacts the pest *Spodoptera frugiperda*. The moths exposed to predator signals showed reduced reproduction and changed metabolism. This supports the idea that fear of predators can suppress pest populations, offering an additional, non-lethal tool for pest management.

Chemical control methods must be carefully integrated with biological control to avoid harming beneficial insects, such as ladybird beetles. Pan et al. [Article 5] tested two commonly used insecticides and found that they negatively affect the feeding behavior and development of *H. axyridis*, even at low doses. These results stress the importance of choosing pesticides that are toxic to natural enemies. In another study, Li et al. [Article 6] looked at how the diet affects ladybird beetles by feeding *Propylea japonica* various prey items, including the eggs of *Ephestia kuehniella*—a commonly used alternative food source. Their findings revealed that *E. kuehniella* eggs provided rich nutrients and low microbial content, which not only supported robust growth but also influenced gene expression. These insights can help improve artificial diets for mass-rearing ladybirds.

Beyond these applied studies, Poorani [Article 7] conducted a review of the relatively understudied subfamily Microweiseinae in India while also describing a new species. This work underscores the necessity of exploring the full diversity of ladybird beetles, as certain lesser-known groups may prove valuable in specific pest control scenarios—particularly in tropical regions.

Taken together, the studies featured in this Special Issue illustrate how research encompassing genes, reproduction, behavior, diet, and diversity is helping to shape the future of ladybird-based biological control. Ladybird beetles exhibit remarkable flexibility and adaptability, and their efficacy can be enhanced through selective breeding, optimized diets, and deliberate integration with other pest control strategies—particularly when guided by robust scientific insights.

## Data Availability

No datasets were generated or analyzed.

